# P2Y6 receptor-mediated signaling amplifies TLR-induced pro-inflammatory responses in microglia

**DOI:** 10.3389/fimmu.2022.967951

**Published:** 2022-09-20

**Authors:** Raissa Timmerman, Ella A. Zuiderwijk-Sick, Jeffrey J. Bajramovic

**Affiliations:** Alternatives Unit, Biomedical Primate Research Centre, Rijswijk, Netherlands

**Keywords:** P2RY6, microglia, bone marrow-derived macrophages, TLR, heat shock proteins, neuroinflammation

## Abstract

TLR-induced signaling initiates inflammatory responses in cells of the innate immune system. These responses are amongst others characterized by the secretion of high levels of pro-inflammatory cytokines, which are tightly regulated and adapted to the microenvironment. Purinergic receptors are powerful modulators of TLR-induced responses, and we here characterized the effects of P2Y6 receptor (P2RY6)-mediated signaling on TLR responses of rhesus macaque primary bone marrow-derived macrophages (BMDM) and microglia, using the selective P2RY6 antagonist MRS2578. We demonstrate that P2RY6-mediated signaling enhances the levels of TLR-induced pro-inflammatory cytokines in microglia in particular. TLR1, 2, 4, 5 and 8-induced responses were all enhanced in microglia, whereas such effects were much less pronounced in BMDM from the same donors. Transcriptome analysis revealed that the overall contribution of P2RY6-mediated signaling to TLR-induced responses in microglia leads to an amplification of pro-inflammatory responses. Detailed target gene analysis predicts that P2RY6-mediated signaling regulates the expression of these genes *via* modulation of the activity of transcription factors NFAT, IRF and NF-κB. Interestingly, we found that the expression levels of heat shock proteins were strongly induced by inhibition of P2RY6-mediated signaling, both under homeostatic conditions as well as after TLR engagement. Together, our results shed new lights on the specific pro-inflammatory contribution of P2RY6-mediated signaling in neuroinflammation, which might open novel avenues to control brain inflammatory responses.

## Introduction

Toll-like receptors (TLR) comprise a family of pattern recognition receptors that are involved in pathogen recognition by innate immune cells in particular (1). For human and non-human primates, ten members of the TLR family have been described (2, 3). TLR activation initiates a cascade of intracellular signaling events that culminate amongst others in the activation of transcription factors nuclear factor (NF)-κB, activator protein (AP)-1 and interferon regulatory factors (IRFs), which in turn induce the expression of inflammatory soluble mediators such as the cytokines interleukin (IL)-1α, IL-6, IL-8, IL-12 and tumor necrosis factor (TNF)-α (4, 5). TLR-induced responses must be strictly regulated since uncontrolled activation can amongst others lead to chronic inflammation (6).

During inflammatory conditions, including TLR activation, extracellular levels of adenosine and other nucleotides, such as uridine diphosphate (UDP), rapidly rise (7, 8). These molecules can trigger autocrine or paracrine signaling through two families of purinergic receptors, P1 and P2 respectively, that are expressed by many types of immune cells including macrophages. The family of P1 receptors includes four subtypes of G protein-coupled adenosine receptors, whereas the family of P2 receptors includes seven subtypes of ATP-selective ligand-gated ion-conducting P2X receptors, and eight subtypes of G-protein-coupled P2Y receptors. Signaling by purinergic receptors can modulate the secretion of cytokines, migration, phagocytosis and apoptosis by the expressing cell (9, 10). It is therefore not surprising that purinergic signaling is involved in the pathophysiology of multiple disorders, such as the neurological diseases Alzheimer’s disease (AD), Parkinson’s disease (PD), Huntington’s disease, amyotrophic lateral sclerosis and multiple sclerosis (11–13). In this context, many studies have reported on the potential of purinergic receptor-mediated signaling to modulate TLR-induced responses in microglia, the resident macrophages of the brain (14–19). For example, in rodents, inhibition of P2Y6 receptor (P2RY6)-mediated signaling by deletion or antagonist approaches reduced lipopolysaccharide (LPS)-induced neuronal loss in the substantia nigra and the production of pro-inflammatory cytokines, respectively (16, 19).

The contribution of P2RY6-mediated signaling to the release of pro-inflammatory cytokines in microglia has been attributed to ERK1/2, calcium/NFAT, MAP kinases and NF-κB activation (16, 20, 21), but the exact mechanisms by which P2RY6-mediated signaling contributes to TLR-induced neuroinflammatory processes remain largely unknown. As studies emphasize the importance of P2RY6-mediated signaling in neuroinflammation, we choose to directly compare the effects of P2RY6-mediated signaling on TLR-induced immune responses in microglia to those in bone marrow-derived macrophages (BMDM). We isolated primary cells from rhesus macaques, outbred animals that are evolutionary close to humans (22, 23), and used the selective P2RY6 antagonist MRS2578 to demonstrate that P2RY6-mediated signaling broadly amplifies the production of TLR-induced pro-inflammatory cytokines in microglia, while such effects were much less pronounced in BMDM. Transcriptome analysis reveals the breadth of the pro-inflammatory contribution of P2RY6-mediated signaling to TLR-induced responses in microglia, and predicts that enhanced activation of the transcription factors NFAT, IRFs and NF-κB is a likely explanation for this. Interestingly, we also observed that P2RY6-mediated signaling strongly inhibits the mRNA expression levels of heat shock proteins (HSP), both under homeostatic conditions as well as after TLR engagement. As this phenomenon was observed both in BMDM as well as in microglia, it is probably not directly related to the P2RY6-mediated amplification of pro-inflammatory responses but may well be relevant when considering the therapeutical use of P2RY6 inhibitors.

## Materials and methods

### Reagents

P2RY6 antagonist N,N’’-1,4-Butanediylbis[N’-(3-isothiocyanatophenyl)thiourea (MRS2578) (Tocris Bioscience, Bristol, UK) was reconstituted in DMSO at a concentration of 50 mM. MRS2578 is a selective -yet not entirely specific- antagonist of P2Y6 nucleotide receptors with an IC50 value of 37 nM at human P2RY6. It displays insignificant activity at P2Y1, P2Y2, P2Y4 and P2Y11 receptors (IC50 > 10 μM). DMSO controls were included in all assays where MRS2578 was used. TLR agonists used were Pam3CSK4 (TLR1/2), LPS (TLR2/4), ultrapure LPS (TLR4), Flagellin (TLR5), and CL075 (TLR8; all In vivogen, San Diego, CA). Used concentrations of the TLR agonists can be found in the figure legends.

### Animals

Brain tissue and bone marrow were obtained from adult rhesus macaques (*Macaca mulatta*) of either sex without neurological disease that became available from the outbred breeding colony or from other studies (all studies were ethically reviewed and approved by the Ministry of Agriculture, Nature and Food Quality of the Netherlands). No animals were sacrificed for the exclusive purpose of the initiation of primary cell cultures. Better use of experimental animals contributes to the priority 3Rs program of the Biomedical Primate Research Centre. Individual identification data of the animals are listed in **Table 1**.

**Table 1:** Individual identification data of rhesus macaques.

Monkey ID nr.	Age (years)	Sex	Weight (kg)	Origin
R00043	21	F	11,6	India
R01085	21	F	6,7	India
R02008	19	M	14,2	India
R02046	19	F	11,2	India
R02060	18	F	8,2	India
R03098	17	F	7,3	India
R06050	15	F	10,9	India
R06054	15	F	7,0	India
R07110	13	M	10,7	India
R12016	9	M	8,1	India
R13169	8	M	15,0	India
R14143	6	F	4,8	India
R15009	6	M	10,0	India
R15143	6	M	6,6	India
R15150	6	M	9,2	India
R17023	5	M	8,0	India
R18015	3	M	4,9	India

### Primary cells isolation and cell culture

Rhesus macaque primary microglia were isolated as described previously (22, 23). In short, Frontal subcortical white matter samples were collected in primary microglia medium (PMM) comprised of 1:1 v/v DMEM (high glucose)/HAM F10 Nutrient mixture (Gibco, Thermo Fisher Scientific, Waltham, MA) supplemented with 10% v/v heat inactivated FBS (TICO Europe, Amstelveen, The Netherlands), 2 mM glutamax, 50 units/mL penicillin and 50 µg/mL streptomycin (all from Gibco). Microglia isolations were initiated from cubes of ~4.5 g tissue that were depleted of meninges and blood vessels manually. Tissue was chopped into cubes of less than 2 mm^2^ using gentleMACS™ C tubes (Miltenyi Biotec, Bergisch Gladbach, Germany) and incubated at 37°C for 20 min in PBS containing 0.25% (w/v) trypsin (Gibco) and 1 mg/mL bovine pancreatic DNase I (Sigma-Aldrich, Saint Louis, MO) and mixed every 5 min. The supernatant was discarded (no centrifugation), the pellet was washed in PMM and passed over a 100 μm nylon cell strainer (Falcon; Becton Dickinson Labware Europe) and centrifuged for 7 min at 524 g. The pellet was resuspended in 22% (vol/vol) Percoll (GE Healthcare Bio-Sciences AB, Uppsala, Sweden), 37 mM NaCl and 75% (vol/vol) myelin gradient buffer (5.6 mM NaH_2_PO_4_, 20 mM Na_2_HPO_4_, 137 mM NaCl, 5.3 mM KCl, 11 mM glucose, 3 mM BSA Fraction V (Sigma-Aldrich), pH 7.4). A layer of 100% myelin gradient buffer was added on top and centrifuged at 1561 g for 30 min (minimal brake). The pellet was washed in PMM and centrifuged for 7 min at 524 g. Cells were plated at a density of 6.5 * 10^4^ cells/cm^2^ in tissue-culture treated well plates (Corning Costar Europe, Badhoevedorp, the Netherlands) in PMM. After overnight incubation at 37°C in a humidified atmosphere containing 5% CO_2_, unattached cells and myelin debris were removed by washing with PBS twice and attached cells were cultured in fresh PMM supplemented with 20 ng/mL macrophage-colony stimulating factor (M-CSF; PeproTech, London, UK). Cells were kept in culture for 8 days without passaging. Half of the medium was replaced by fresh medium containing M-CSF every 3-4 days.

Rhesus macaque primary bone marrow-derived macrophages were isolated by flushing the bone marrow from the femur (~4 cm) with PBS, followed by passing the suspension over a 100 μm nylon cell strainer (Falcon) and density gradient centrifugation using Lymphoprep (Axis Shield PoC AS, Oslo, Norway) according to manufacturer’s protocol. Cells were plated at a density of 1.3 * 10^5^ cells/cm^2^ in tissue-culture treated well plates (Corning Costar Europe) in RPMI 1640 (Gibco) supplemented with 10% v/v heat inactivated FBS (Tico), 2 mM glutamax, 50 units/ml penicillin, 50 μg/ml streptomycin (all Gibco) and 20 ng/ml M-CSF (Peprotech). Half of the medium was replaced by fresh medium containing M-CSF at day 4. Cells were kept in culture for 8 days without passaging.

### Knockdown of P2RY6 in primary microglia

Transfection of siRNAs in adult rhesus macaque primary microglia was performed using the Glial-Mag kit (OZ Biosciences, San Diego, CA) as described by Carrillo-Jimenez and colleagues (24), with a few modifications. The described method here refer to a 24-well plate format. For one well, 72 nM siRNA (Horizon Discovery, Waterbeach, UK) was added to a microcentrifuge tube (for siP2RY6, 18 nM of each siRNA was used). 100 µl DMEM (Gibco) without supplements was added to the tube with siRNA and mixed by vortexing. The contents of this tube were added to a new microcentrifuge tube containing 0.6 µl Glial-Mag (OZ Biosciences) and mixed gently by pipetting up and down five times. The mixture was incubated for 20 min at room temperature. 100 µl culture medium was removed from the well to assure a final volume of 400 µl. 100 µl siRNA + Glial-Mag mixture was added drop by drop to the well. Subsequently, 5 µl Glial-Boost (100x) (OZ Biosciences) was added to the well. To ensure even distribution, the culture plate was moved back and forward a few times. The culture plate was placed on top of the magnetic plate (provided by the Glial-Mag kit) inside the cell incubator for 30 min. The magnetic plate was removed, and the culture plate was placed in the incubator for 3 more hours at 37°C. After 3 h incubation, culture medium was replaced for fresh PMM medium containing 20 ng/mL M-CSF. Stimulation experiments with 10 µg/mL uLPS were performed 24 h after the medium change. The different siRNAs used for this study were siGLO Green Transfection Indicator (#D-001630-01-05), ON-TARGETplus Non-targeting Pool (D#001810-10-05) and 4x Custom ON-TARGETplus P2RY6 siRNA specifically designed for rhesus macaques (all from Horizon Discovery). All siRNAs were reconstituted in 1x siRNA Buffer (Horizon Discovery). The sequences of the different siRNAs are provided in **Table 2**.

**Table 2:** Sequences of the different small interfering RNAs (siRNAs).

siRNA	Sequence
siRNA non-targeting (1)	UGGUUUACAUGUCGACUAA
siRNA non-targeting (2)	UGGUUUACAUGUUGUGUGA
siRNA non-targeting (3)	UGGUUUACAUGUUUUCUGA
siRNA non-targeting (4)	UGGUUUACAUGUUUUCCUA
siP2RY6 (1)	UUACGCUGGAUGCCUGUGGUU
siP2RY6 (2)	UUUGGCUGUGAGUUUCUGUUU
siP2RY6 (3)	AGUCGCUUGAAGUUCUCGCUU
siP2RY6 (4)	UAGCGCUGGAAGCUGAUGCUU

### RNA isolation and quantitative RT-PCR

Total cellular RNA was isolated using the RNeasy minikit (Qiagen GmbH, Hilden, Germany) according to manufacturer’s protocol. Subsequently, mRNA was reverse transcribed into cDNA using the RevertAid First Strand cDNA synthesis kit according to the manufacturer’s protocol (Fermentas; Thermo Fisher Scientific). RT-PCRs were performed on the CFX96™ Real-time PCR detection system (Bio-rad Laboratories, Hercules, CA) using primer (Invitrogen; Thermo Fisher Scientific) and probe (human Exiqon probe library, Roche, Woerden, The Netherlands) combinations listed in **Table 3**, and iTaq Universal Probes Supermix (Bio-rad Laboratories). Relative gene expression was standardized to ACTB using the Pfafll method (25).

**Table 3:** Overview of primer/probe combinations used for RT-PCR.

Gene name	Forward primer (5’-3’)	Reverse primer (5’-3’)	Probe
ACTB	GCCCAGCACGATGAAGAT	CGCCGATCCACACAGAGTA	AGGAGGAG
CRYAB	ATGGCAAACATGAAGAGCGC	GTAATGGTGAGAGGGTCCACA	TCTCCAGG
DNAJA4	TTTCAGATCCAAAGAAAAGGGATATTT	ATGTCCATGGGTGAAGAGAAGCTG	GCTGCCTG
DNAJB1	CATCGAAGTGAAGAAGGGGTG	AACAAAGACGATATCAGCTGGAA	GGAAGGAG
HSP90AA1	CATGAAGACTCACAAAATCGGAAG	TTTCCTTCATTCTGGTGCAGT	CTGCCTCT
HSPA5	TCCGGTCTACTATGAAGCCC	AATTCGAGTCGAGCCACCAA	CTTCCAGC
HSPB1	CGGACGAGCTAACGGTTAAG	GTGAAGCACCGGGAGATGTAG	CTCCACCA
HSPD1	GTTGGTCTTCAGGTTGTGGC	CTCCAAACACTGCACCACCA	CCTGGAGC
HSPH1	CTCACAGTCTCCCCCTTCAC	GGCAGCTCGACATTCACCA	GGAGGCTG
IL-1α	AATAACCTGGAGGCCATCG	GCTAAAAGGTGCCGACCTG	CAGAGGAA
IL-6	ACAAAAGTCCTGATCCAGTTCC	GTCATGTCCTGCAGCCACT	CAGCAGGC
IL-8	TCTGTGTAAACATGACTTCCAAGC	CACTCCTTGGCAAAACTGC	CAGAGGAA
IL-12p40	CCACATTCCTACTTCTCCCTGA	ACCGTGGCTGAGGTCTTGT	TCCAGGTC
P2RY6	AGCTGTCTTTGCTGCCACA	GGGCTGAGGTCATAGCAAAC	GCTGGATG
TNF-α	AAGCCTGTAGCCCATGTTGT	GCTGGTTATCTGTCAGCTCCA	CCAGGAGG

### Cytokine analysis

Old world monkey sandwich ELISA kits for IL-6, IL-8, IL-12p40 and TNF-α, (U-CyTech, Utrecht, The Netherlands) were used for quantification of the cytokines in cell culture supernatants according to manufacturer’s instructions. Cytokine levels were analyzed using the ELx800™ Absorbance Microplate Reader (Biotek, Winooski, VT).

### Next generation RNA sequencing

The NEBNext Ultra II Directional RNA Library Prep Kit for Illumina (New England Biolabs, Ipswich, MA) was used to prepare and process the total RNA samples. Briefly, mRNA was isolated from total RNA using oligo(dT) magnetic beads. After fragmentation of the mRNA, cDNA synthesis was performed followed by ligation of sequencing adapters and PCR amplification. The quality and yield after sample preparation were measured with a fragment analyzer (Agilent Technologies, Amstelveen, The Netherlands). Clustering and sequencing using the Illumina NovaSeq 6000 was performed according to manufacturer’s protocols. Prior to alignment, the reads were trimmed for adapter sequences using fastp v0.20 (26), using default parameters. The *Macaca mulatta* genomic reference (Mmul_10) was used for alignment of the reads for each sample. The reads were mapped to the reference sequence using a short-read aligner based on Burrows-Wheeler Transform (STAR2 v2.5.4) with default settings. SAMtools v1.10 package (http://htslib.org/, RRID : SCR_002105) was used to sort and index the BAM files. Based on the mapped locations in the alignment file the frequency of how often a read was mapped on a transcript was determined with HTSeq v0.11.0 (https://htseq.readthedocs.io/en/release_0.11.1/, RRID : SCR_005514). Only unique reads that fall within exon regions were counted. The counts were saved to count files, which were served as input for downstream RNA sequencing analysis.

### Cell viability assay

To assess the cytotoxicity of MRS2578 on primary bone marrow-derived macrophages and microglia, cell viability was investigated using the live/dead viability/cytotoxicity kit (Thermo Fisher Scientific). In short, cells were rinsed twice with PBS. Subsequently, 2 µM of calcein AM and 4 µM of ethidium homodimer-1 (both part of the live/dead assay kit) in PBS were added to each well and incubated for 45 min at room temperature protected from light. Samples were rinsed with PBS and the nuclei were stained by incubation with 1 µM Hoechst 33342 (Thermo Fisher Scientific) in PBS for 10 min at room temperature. Samples were rinsed with PBS and fixed with 2% paraformaldehyde in PBS (Affymetrix, Santa Clara, CA) for 30 min at room temperature. Samples were rinsed twice with PBS and live and dead cells were visualized using a Leica DMI6000 fluorescence microscope and LASX software.

### TUNEL assay

To label fragmented DNA of apoptotic cells, the DeadEnd Fluorometric TUNEL System kit (Promega, Madison, WI) was used according to the manufacturer’s protocol. Briefly, cells grown on coverslips were fixed in 2% paraformaldehyde solution in PBS for 30 min at room temperature, rinsed twice with PBS and then treated with 0.2% Triton X-100 in PBS for 5 min at room temperature. To generate positive controls, the samples were incubated in DNase I buffer (40 mM Tris-HCl (pH 7.9), 10 mM NaCl, 6 mM MgCl_2_ and 10 mM CaCl_2_) for 5 min and treated with DNase I (± 6 units/mL; Qiagen GmbH) in DNase I buffer for 10 min. After two washes with PBS, samples were incubated in equilibration buffer (part of the TUNEL assay kit) for 10 min at room temperature. The reaction mix was prepared according to manufacturer’s protocol. Staining was carried out for 1 h at 37°C in a humidified chamber protected from light. The reaction was arrested by triple incubation in 2x SSC (part of the TUNEL assay kit) for 5 min. Samples were rinsed twice with PBS and mounted using ProLong™ Diamond Antifade + DAPI (Thermo Fisher Scientific). Images were acquired using a Leica DMI6000 fluorescence microscope and LASX software.

### Bioinformatics

BiomaRt Bioconductor Package (https://bioconductor.org/packages/release/bioc/html/biomaRt.html, RRID : SCR_019214) was used to annotate the genes and to generate a gene symbol list (27, 28). The accession number for the RNA-sequencing data from cultured primary microglia from rhesus macaques exposed to 10 µg/mL uLPS in the presence or absence of 5 µM P2RY6 antagonist MRS2578 reported in this paper is GSE195866. Data were inspected using principal component analysis and heatmaps generated with heatmap.2 of Bioconductor package gplots. Differential gene expression analysis was performed with Bioconductor package EdgeR (https://bioconductor.org/packages/release/bioc/html/edgeR.html, RRID : SCR_012802) (29). The Molecular Signatures Database (MsigDB, http://software.broadinstitute.org/gsea/msigdb/index.jsp, RRID : SCR_016863) was used to perform gene ontology analysis, canonical pathway analysis and transcription factor target analysis (30, 31).

### Statistics

GraphPad Prism 9.2.0 (GraphPad Software, San Diego, CA) was used for statistical analysis. Statistical details of experiments can be found in the figure legends.

## Results

### Microglia are particularly sensitive to P2RY6-mediated amplification of the production of TLR-induced pro-inflammatory cytokines

**Figure 1:**
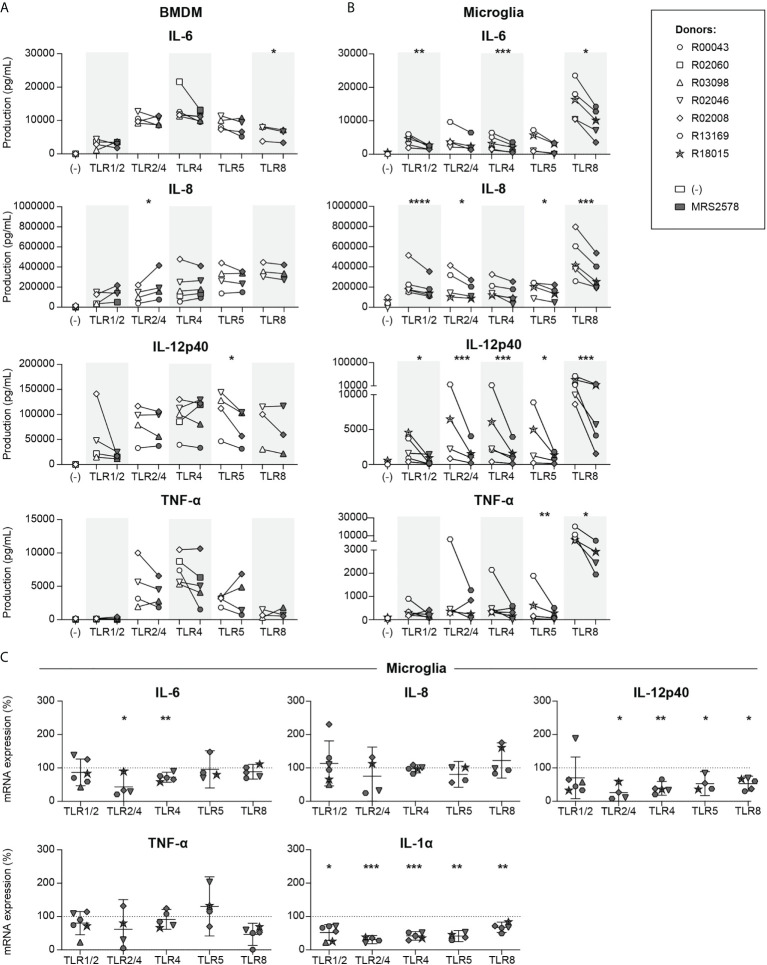
P2RY6-mediated signaling is broadly involved in microglia TLR-induced pro-inflammatory cytokine production and mRNA expression. Primary bone marrow-derived macrophages (BMDMs) and microglia from rhesus macaques were exposed for 16 h to different TLR ligands in the absence (white symbols) or presence (grey symbols) of 1 h pre-incubation of 5 µM P2RY6 antagonist MRS2578. TLR ligands used were 1 µg/mL PAM_3_CSK_4_ (TLR1/2), 10 ng/mL LPS (TLR 2/4), 10 ng/mL ultrapure LPS (TLR4), 100 ng/mL Flagellin (TLR5) or 1 µg/mL CL075 (TLR8). IL-6, IL-8, IL-12p40 and TNF-α production levels of **(A)** BMDM and **(B)** microglia are shown in pg/mL. Symbols represent different donors. n=3-5, paired t-test on log-transformed data, *p < 0.05, **p < 0.01, ***p < 0.005, ****p < 0.001. **(C)** Microglia mRNA expression levels of pro-inflammatory cytokines in the presence of 5 µM MRS2578 are expressed relative to mRNA expression after exposure to each TLR ligand alone (dotted line = 100%). Symbols represent different donors. Horizontal lines indicate mean values with 95% confidence intervals. n=4-6, paired t-test on log-transformed data, *p < 0.05, **p < 0.01, ***p < 0.005.

It has been reported that P2RY6-mediated signaling modulates LPS (TLR2/4)-induced cytokine responses in resident macrophages (16, 32), but studies that compare modulation of such responses in different subpopulations of macrophages are lacking. We therefore analyzed the involvement of P2RY6-mediated signaling on pro-inflammatory cytokine production as induced by a broad range of TLR agonists in primary BMDM and microglia from rhesus macaques. Engagement of TLR1/2 (by Pam_3_CSK_4_), TLR2/4 (by LPS), TLR4 (by ultrapure (u)LPS), TLR5 (by Flagellin), and TLR8 (by CL075) potently induced IL-6, IL-8 and IL-12p40 production in both cell types (**Figures 1A, B**), while exposure to TLR1/2 agonist induced the production of TNF-α in microglia only. Exposure of BMDM to the selective P2RY6 antagonist MRS2578 inhibited the production of TLR8-induced IL-6 and TLR5-induced IL-12p40, whereas TLR2/4-induced IL-8 was enhanced (**Figure 1A**). By comparison, exposure of microglia to MRS2578 had much more potent effects and reduced the TLR1/2-, TLR4- and TLR8-induced production of IL-6, the TLR1/2-, TLR2/4-, TLR5- and TLR8-induced production of IL-8, the TLR1/2-, TLR2/4-, TLR4-, TLR5- and TLR8-induced production of IL-12p40, and the TLR5- and TLR8-induced production of TNF-α (**Figure 1B**). Mean log fold changes of **Figures 1A, B** are presented in **Table S1**. Of note, we intended to analyze IL-1α production as well, but this was not possible since commercial ELISA reagents are not available for non-human primates. Inhibition of TLR-induced cytokine responses could not be attributed to MRS2578-associated cytotoxicity. Exposure to up to 5 µM MRS2578 did not affect the numbers of viable cells, neither for BMDM nor for microglia (**Figure S1**). Only when cells were exposed to concentrations as high as 25 µM MRS2578, decreases in cell viability were observed. We next questioned whether the differential sensitivity to P2RY6-mediated signaling between BMDM and microglia could be attributed to different P2RY6 mRNA expression levels, but these were comparable between BMDM and microglia (**Figure S2**).

To investigate whether the reduced production levels of TLR-induced pro-inflammatory cytokines in microglia in the presence of P2RY6 antagonist were correlated to reduced mRNA expression levels we used a real-time PCR approach. Engagement of different TLRs potently induced the mRNA expression levels of IL-6, IL-8, IL-12p40, TNF-α and IL-1α (**Table S2**), and exposure to P2RY6 antagonist significantly reduced the mRNA expression levels of TLR2/4- and TLR4-induced IL-6 (**Figure 1C**). In addition, TLR2/4-, TLR4-, TLR5- and TLR8-induced IL-12p40 and TLR1/2-, TLR2/4-, TLR4-, TLR5- and TLR8-induced IL-1α mRNA expression levels were also significantly reduced in the presence of P2RY6 antagonist. Although we observed some minor discrepancies between production (ELISA) and mRNA expression (RT-PCR) levels, the overall data clearly demonstrate that P2RY6-mediated signaling broadly affects TLR-induced pro-inflammatory cytokine production and mRNA expression levels, in microglia in particular.

**Figure 2:**
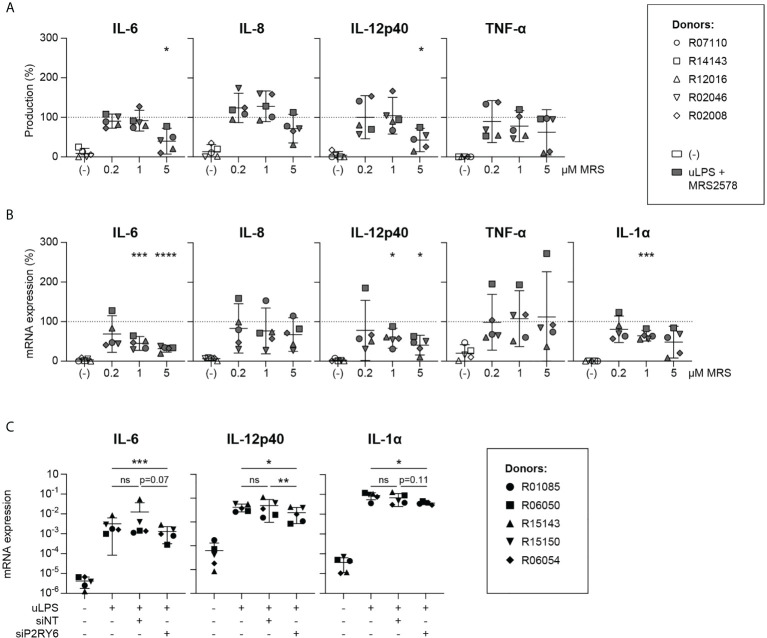
uLPS-induced IL-6, IL-12p40 and IL-1α expression levels are dependent on P2RY6-mediated signaling. Primary microglia from rhesus macaques were exposed for 16 h to 10 µg/mL uLPS (TLR4) with or without 1 h pre-incubation of 0.2, 1 or 5 µM P2RY6 antagonist MRS2578. The effects of P2RY6 antagonist on uLPS-induced IL-6, IL-8, IL-12p40, TNF-α and IL-1α were analyzed. Graphs show the effects of P2RY6-mediated signaling on TLR4-induced **(A)** cytokine production and **(B)** mRNA expression levels. Levels of all graphs are expressed relative to levels after exposure to uLPS alone (dotted line =100%). Symbols represent different donors. Horizontal lines indicate mean values with 95% confidence intervals. n=5, paired t-test on log-transformed data, *p < 0.05, ***p < 0.005, ****p < 0.001. **(C)** Effects of P2RY6 knockdown (siP2RY6) on uLPS-induced IL-6, IL-12p40 and IL-1α mRNA expression levels. siNT = transfection with non-targeting control siRNA. Symbols represent different donors. n=5, one-way ANOVA on log-transformed data, ns, not statistically significant. *p < 0.05, **p < 0.01, ***p < 0.005.

To further characterize the effects of P2RY6-mediated signaling on TLR-induced responses, we continued with the robust and specific TLR4 agonist uLPS. Microglia were stimulated with uLPS in the presence of escalating concentrations of MRS2578. Exposure to uLPS strongly induced the production of IL-6, IL-8, IL-12p40 and TNF-α (**Table S3**), whereas exposure to MRS2578 inhibited the uLPS-induced production of IL-6 and IL-12p40 in a dose-dependent manner (**Figure 2A**). This was confirmed at the transcription level. IL-6, IL-8, IL-12p40, TNF-α and IL-1α mRNA expression levels were strongly induced after uLPS exposure (**Figure S3**), and in the presence of MRS2578, uLPS-induced mRNA gene expression levels of IL-6, IL-12p40 and IL-1α were dose-dependently inhibited (**Figure 2B**).

To confirm that our results were attributable to the selective inhibition of P2RY6-mediated signaling, we silenced the expression of P2RY6 in microglia with P2RY6-targeting siRNAs and analyzed IL-6, IL-12p40 and IL-1α mRNA expression levels after exposure to uLPS. P2RY6 knockdown decreased P2RY6 mRNA expression by ± 15-fold as confirmed by RT-PCR (**Figure S4**, **Table S4**). In comparison to untransfected microglia or to microglia that were transfected with non-targeting control siRNAs, uLPS-induced IL-12p40 mRNA levels in microglia transfected with P2RY6-targeting siRNAs were significantly inhibited (**Figure 2C**, **Table S4**). In addition, TLR4-induced IL-1α and IL-6 mRNA levels were also reduced, although not significantly. Mean log fold change mRNA expression values of **Figure 2C** are presented in **Table S5**.

### RNA transcriptome analysis reveals that P2RY6-mediated signaling broadly amplifies uLPS-induced pro-inflammatory responses

**Figure 3:**
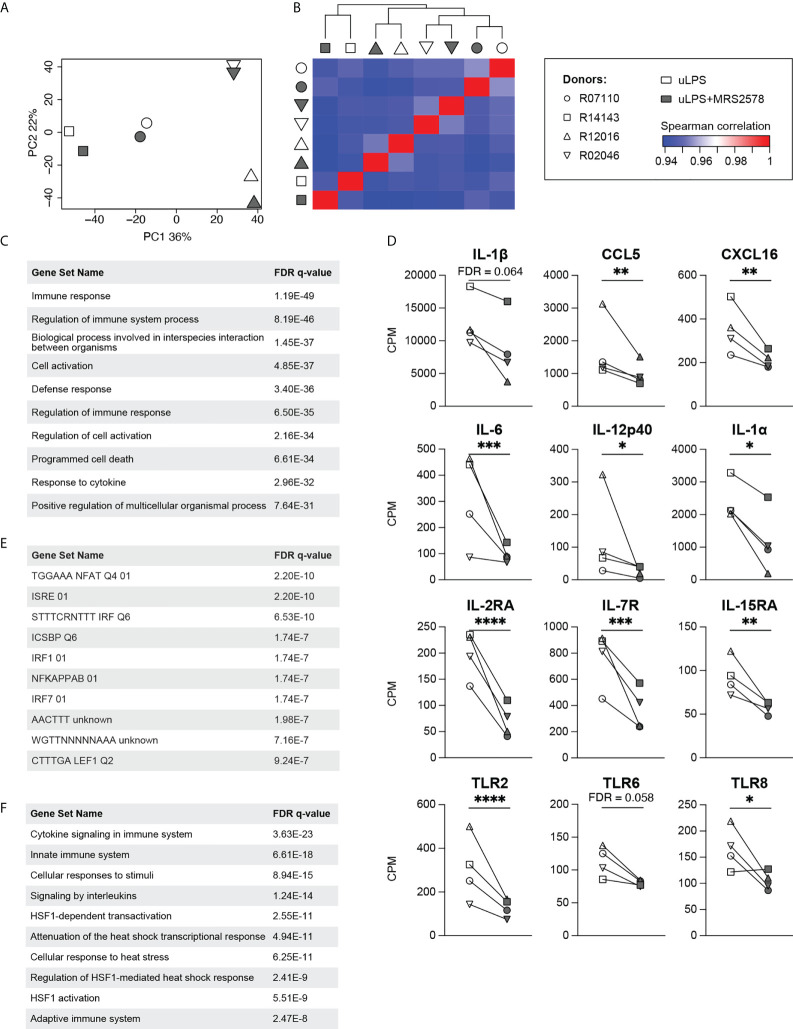
Effects of P2RY6-mediated signaling on the transcriptomes of uLPS-exposed primary microglia. **(A)** Principal component analysis and **(B)** Spearman correlation heatmap of the transcriptomes of primary microglia from four adult rhesus macaques exposed to 10 µg/mL uLPS (TLR4), with (grey symbols) or without (white symbols) 1 h pre-incubation with 5 µM P2RY6 antagonist MRS2578. n=4, symbols represent different donors. **(C)** Biological processes associated with the 302 differentially expressed genes were analyzed using the molecular signatures database (MSigDB). FDR = false discovery rate. **(D)** Gene expression levels (CPM) of several genes associated with immune responses. Microglia were exposed to uLPS, with (grey symbols) or without (white symbols) 1 h pre-incubation with MRS2578. n=4, EdgeR false discovery rates (FDR) are used to display statistical differences, *FDR < **FDR < 0.01, ***FDR < 0.005, ****FDR < 0.001. **(E)** Transcription factor target analysis of the downregulated genes in the presence of P2RY6 antagonist analyzed using MSigDB. FDR = false discovery rate. **(F)** Canonical pathways associated with the 302 differentially expressed genes were analyzed using MSigDB. FDR = false discovery rate.

To get a broader overview of the P2RY6-mediated effects, we compared the RNA transcriptomes of microglia exposed to the TLR4 agonist uLPS in the presence of MRS2578 to those exposed to uLPS only. Of note, for this analysis we selected four donors that were also used in the previous section (**Figure 2**). Principal component analysis and heatmap analysis show that the transcriptomes from individual donors cluster together, rather than samples that were exposed to similar stimulation conditions (**Figures 3A, B**). Such large donor-donor variability is not uncommon when working with samples derived from outbred individuals (22). Nevertheless, differential gene expression analysis demonstrates that 302 gene products were expressed at significantly different levels when cells were exposed to P2RY6 antagonist (FDR < 0.05; **Table S6**). Gene ontology analysis showed that the differentially expressed genes (DEG) are associated with biological processes such as immune response (93 genes), defense response (76 genes) and response to cytokine (55 genes; **Figure 3C**). It should be noted that only the top 10 biological processes are presented and that genes can be associated with multiple biological processes. The DEG associated with each biological process can be found in **Table S7**. Indeed, transcripts encoding for cytokine IL-1β (not significant) and the chemokines CCL5 and CXCL16 (both significant) were decreased when P2RY6-mediated signaling was inhibited (**Figure 3D**). Importantly, transcriptome analysis confirmed the downregulation of IL-6, IL-12p40 and IL-1α mRNA expression levels in the presence of P2RY6 antagonist. In addition to cytokine and chemokine transcripts, we observed that IL-2, IL-7 and IL-15 receptor transcripts were also decreased in the presence of P2RY6 antagonist. Furthermore, TLR6 (not significant), TLR2 and TLR8 (both significant) expression levels were also markedly reduced. These data demonstrate the broad pro-inflammatory contribution of P2RY6-mediated signaling to uLPS-induced responses.

In order to gain insight into the molecular underpinnings of the P2RY6-mediated effects on uLPS-induced responses, we analyzed which transcription factor targets were shared between the downregulated genes. This analysis identifies NFAT, IRFs and NF-κB, as potential modulated targets by P2RY6-mediated signaling (**Figure 3E**). Of note, only the top 10 transcription factor targets are presented. The downregulated genes associated with the transcription factor targets listed in **Figure 3E** can be found in **Table S8**. The possible involvement of NF-κB is in line with the notion that the promoter regions of IL-1α, IL-6 and IL-12p40 all share binding sites for NF-κB (33–35). Finally, we performed a canonical pathway analysis of the 302 DEG using the Reactome Pathway Database. Besides cytokine and interleukin signaling pathways (42 genes), we unexpectedly observed that multiple heat shock transcription factor 1 (HSF1)-mediated pathways (12 genes) were present in this list (**Figure 3F**). Again, only the top 10 pathways are presented. The DEG associated with the top 10 pathways are displayed in **Table S9**.

### Inhibition of P2RY6-mediated signaling induces the expression of heat shock protein genes

**Figure 4:**
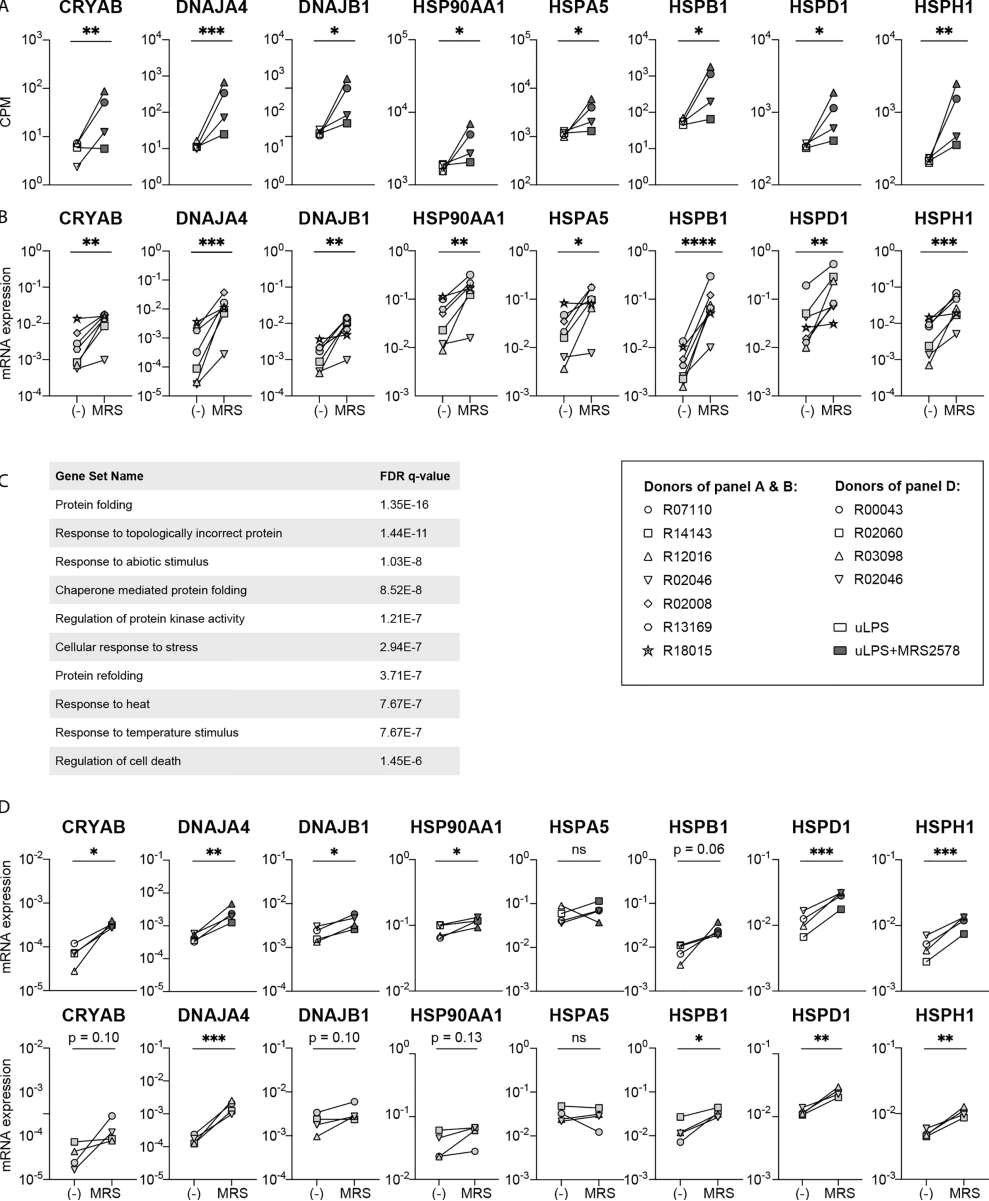
Exposure to P2RY6 antagonist MRS2578 induces the expression of heat shock protein genes. **(A)** Gene expression levels (CPM) of heat shock protein genes in microglia exposed to uLPS (TLR4), with (grey symbols) or without (white symbols) 1 h pre-incubation with MRS2578. n=4, EdgeR false discovery rates (FDR) are used to display statistical differences, * FDR < 0.05 ** FDR < 0.01 *** FDR < 0.005. **(B)** mRNA expression levels measured by RT-PCR of heat shock protein genes in homeostatic microglia and microglia exposed to 5 µM MRS2578. Symbols represent different donors. n=7, paired t-test on log-transformed data, *p < 0.05, **p < 0.01, ***p < 0.005, ****p < 0.001. **(C)** Biological processes associated with upregulated differentially expressed genes in uLPS exposed microglia in the presence of P2RY6 antagonist. FDR = false discovery rate. **(D)** mRNA expression levels measured by RT-PCR of heat shock protein genes in homeostatic BMDM and BMDM exposed to 5 µM MRS2578, either in the absence or presence of 10 ng/mL uLPS. Symbols represent different donors. n=4, paired t-test on log-transformed data, ns, not statistically significant, *p < 0.05, **p < 0.01, ***p < 0.005.

To our surprise, transcriptome analysis revealed that inhibition of P2RY6-mediated signaling in uLPS-exposed microglia led to a strong induction of the expression levels of multiple heat shock protein (HSP) genes, including CRYAB, DNAJA4, DNAJB1, HSP90AA1, HSPA5, HSPB1, HSPD1 and HSPH1 (**Figure 4A**). Interestingly, RT-PCR analysis showed that these genes were also upregulated in microglia exposed to P2RY6 antagonist alone (**Figure 4B**), suggesting a role for homeostatic P2RY6-mediated signaling in microglia. We performed a gene set enrichment analysis of the upregulated genes in uLPS-exposed microglia in the presence of P2RY6 antagonist and found that these genes were associated with biological processes such as protein folding, response to topologically incorrect protein and chaperone mediated protein folding, (**Figure 4C**), which are all well described biological processes linked to HSP functions. The upregulation of HSP might indicate that the absence of P2RY6-mediated signaling results in cellular stress, as is also suggested by the DEG associated with programmed cell death in the presence of P2RY6 antagonist (**Figure 3C**). To analyze this in more detail, we performed TUNEL assays and observed that exposure to MRS2578 did not induce apoptosis in microglia (**Figure S5**). Even in combination with exposure to uLPS, the percentage of apoptotic microglia remained very low (<0.4%).

Finally, we questioned whether the amplification of pro-inflammatory responses and the inhibition of HSP expression levels by P2RY6-mediated signaling were related. If this were the case, one might expect that exposure of BMDM to P2RY6 antagonist might induce HSP expression levels to a lesser extent, as the pro-inflammatory effects of P2RY6-mediated signaling were much less pronounced in this cell type. We however observed that inhibition of P2RY6-mediated signaling in BMDM induced the expression levels of HSP genes to a similar extent as in microglia, both under homeostatic conditions as well as after TLR4 engagement (**Figure 4D**).

## Discussion

There is ample evidence for the involvement of P2RY6-mediated signaling in a broad range of central nervous system (CNS) disorders [reviewed in (36)]. While the role of microglial P2RY6 as an essential regulator of phagocytosis in the brain has been widely acknowledged (37, 38), P2RY6-mediated signaling in microglia can also affect cytokine and chemokine production (16, 20). In the present study we demonstrate that P2RY6-mediated signaling broadly amplifies TLR-induced pro-inflammatory signaling in microglia in particular. Modeling predicts that the transcription factors NFAT, IRFs and NF-κB are involved. Furthermore, we describe for the first time a prominent role for P2RY6-mediated signaling in the regulation of HSP gene expression levels.

Engagement of TLRs induced the mRNA expression levels and protein production of multiple pro-inflammatory cytokines and chemokines in both BMDM and microglia. When directly compared to BMDM, we find that in microglia P2RY6-mediated signaling is much more broadly involved in TLR-induced pro-inflammatory cytokine production. These cell type-specific differences are not attributable to differences in P2RY6 mRNA expression levels between BMDM and microglia, as these were comparable. Possibly, differences in P2RY6-mediated responses between different macrophage populations might rather be associated with tissue-specific adaptations of regulatory circuits. Alternatively, microglia might release higher levels of UDP upon TLR exposure when compared to BMDM, resulting in enhanced P2RY6-mediated signaling. Differences in innate immune responses between macrophage populations, including BMDM and microglia has been reported earlier (14, 39) and the impact of ‘nature and nurture’ on BMDM and microglia innate immune responses has been the subject of many studies (40, 41). Our data show that, particularly in the CNS, P2RY6-mediated signaling in microglia most likely amplifies pro-inflammatory responses during inflammation.

The prominent contribution of P2RY6 to pro-inflammatory signaling was further characterized in microglia that were exposed to the TLR4 agonist uLPS and to escalating concentrations of P2RY6 antagonist MRS2578. MRS2578 is a selective -yet not entirely specific- antagonist of P2RY6, but the concentrations used in this study were well below reported IC_50_ values for other P2Y receptors. Whereas TLR4-induced IL-6, IL-12p40 and IL-1α responses were inhibited in the presence of P2RY6 antagonist, IL-8 and TNF-α responses were unaffected. This contrasts with data from a recent study. When mice microglia were exposed to a TLR2/4 agonist in the presence of P2RY6 antagonist, MIP-2 (the murine equivalent of human IL-8) and TNF-α were inhibited. Differences in species, P2RY6 protein-coding sequences (88% overlap between rhesus macaque and mouse), TLR agonists used or differences in sampling timepoints might have been responsible for these discrepant findings. We confirmed the involvement of P2RY6 in TLR4-induced IL-12p40 responses by silencing of P2RY6 gene expression with P2RY6-targeting siRNAs. Although this also affected TLR4-induced IL-1α and IL-6 responses, this was not significant. Transcriptome analysis revealed that the overall contribution of P2RY6-mediated signaling to TLR4-induced responses was of pro-inflammatory nature, as multiple pro-inflammatory cytokines, chemokines and receptors were downregulated in the presence of P2RY6 antagonist. Whether P2RY6-mediated signaling also affected baseline levels of these target genes could not be concluded, as data from unexposed microglia is lacking. Transcription factor target analysis predicted the implication of NF-κB as a target of P2RY6-mediated signaling, which is supported by the notion that the promoter regions of IL-1α, IL-6 and IL-12p40 all share binding sites for NF-κB. Furthermore, NFAT and IRFs were also predicted to be affected by P2RY6-mediated signaling, warranting further detailed biochemical investigations.

Striking was the strong induction of HSP genes in the presence of P2RY6 antagonist, both in uLPS-exposed and in homeostatic microglia. HSPs are a large family of molecular chaperones that are induced by various stressors to provide protection against cellular damage by aiding the folding and assembly of proteins and to prevent protein aggregation (42, 43). The upregulation of multiple HSPs suggests that ablation of P2RY6-mediated signaling induces a stress response in microglia. Since exposure to DMSO (as a solvent control) did not induce the expression of HSP (data not shown), the effect is directly attributable to exposure to the P2RY6 antagonist MRS2578. Noteworthy is that we could not find evidence for MRS2578-induced apoptosis. Although MRS2578 is a potent inhibitor of P2RY6-mediated signaling, it should be tested whether MRS2578 has any off-target effects on microglia to further confirm and elucidate our findings.

HSPs have both pro- and anti-inflammatory effects depending on the cellular location of HSPs and the activation state of the cell [reviewed in (43, 44)]. One could speculate that P2RY6-mediated signaling modulates pro-inflammatory cytokine responses *via* the regulation of HSPs. However, as HSPs were also upregulated in BMDM in the presence of P2RY6 antagonist – and P2RY6-mediated signaling had little effects on TLR-induced pro-inflammatory cytokine responses in this cell type – we have no data to support this hypothesis.

HSPs, including HSP90AA1, HSPA8 and DNAJB1 (all upregulated in the presence of P2RY6 antagonist) are key players in chaperone-mediated autophagy (CMA), a form of autophagy that is impaired in aging and neurodegenerative diseases (45). This suggests that P2RY6-mediated signaling is involved in CMA activity. Functional assays to measure CMA activity are needed to confirm this, but technical limitations associated with the use of primary cells render such assays very challenging (46).

**Figure 5:**
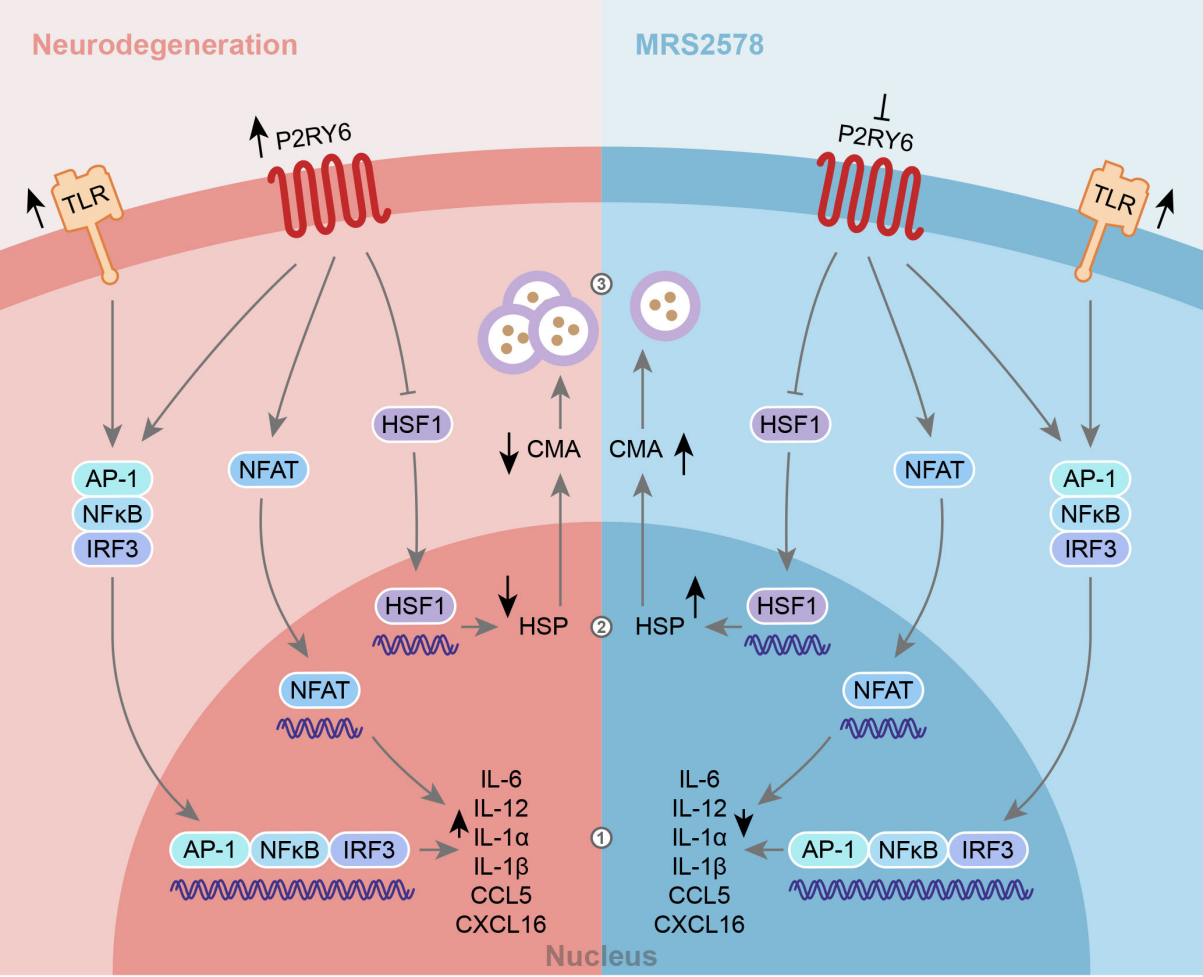
Schematic model of the contribution of P2RY6-mediated signaling during neuroinflammation. During neurodegeneration, TLRs and P2RY6 are described to be upregulated (16, 47, 48). The upregulation of both TLRs and P2RY6 may contribute to 1) chronic neuroinflammation, 2) downregulation of HSP, and 3) autophagosome accumulation due to impaired chaperone-mediated autophagy (CMA) processes, which are all hallmarks of neurodegenerative diseases. Our data show that blocking of P2RY6 with P2RY6 antagonist MRS2578 can 1) reduce the expression of pro-inflammatory cytokines and chemokines and 2) induce the expression of HSPs, which 3) may lead to increased CMA activity.

Taken together, our data suggest that during homeostasis, tonic P2RY6-mediated signaling is a requirement for healthy microglia. Although the protocol and culture medium we used in this study have been characterized extensively to yield microglia that we would characterize as ‘neutral’, it should be taken into account that *in vitro* models for microglia always affect their phenotype and modeling homeostatic microglia remains a major challenge. Our data further suggests that under inflammatory conditions, P2RY6-mediated signaling amplifies TLR-induced pro-inflammatory responses supporting the idea that upregulation of P2RY6, as amongst others seen in neurodegenerative diseases, including AD and PD (16, 47), might be partly responsible for excessive neuroinflammatory responses. In accordance with that idea, previous studies have shown that blocking of P2RY6 is beneficial in models of AD, PD and ischemic stroke (16, 18, 19, 38). Based on our data and published literature, we propose that blocking of P2RY6-mediated signaling can indeed reduce neuroinflammation and might induce HSP expression and CMA activity (**Figure 5**), which are all considered advantageous during neurodegenerative diseases (49–52). As P2RY6 is involved in multiple physiological and pathological cell functions, it should be noted that blocking of P2RY6-mediated signaling may induce both protective and harmful responses depending on the timing, dose and cellular target. Therefore, future research is pivotal to gain further insight in the diverse roles of P2RY6 during neuroinflammatory and neurodegenerative diseases and to determine how and in which context this pathway can be targeted.

## Data availability statement

The datasets presented in this study can be found in online repositories. The names of the repository/repositories and accession number(s) can be found below: https://www.ncbi.nlm.nih.gov/geo/, GSE195866.

## Ethics statement

Brain tissue and bone marrow were obtained from adult rhesus macaques (Macaca mulatta) of either sex without neurological disease that became available from the outbred breeding colony or from other studies (all studies were ethically reviewed and approved by the Ministry of Agriculture, Nature and Food Quality of the Netherlands). No animals were sacrificed for the exclusive purpose of the initiation of primary cell cultures. Better use of experimental animals contributes to the priority 3Rs program of the Biomedical Primate Research Centre.

## Author contributions

Conceptualization, RT and JB. Experimental work, RT and EZ-S. Data analysis, RT, EZ-S, and JB. Manuscript, RT and JB. All authors contributed to the article and approved the submitted version.

## Funding

This study was performed using internal funding only.

## Acknowledgments

We thank E. Remarque for help with the statistical analyses, F. van Hassel for help with the graphical representations of the research, T. Haaksma and I. Kondova for help with the obductions and preparation of CNS material, and H. van Noort and B. ‘t Hart for critical feedback on the manuscript.

## Conflict of interest

The authors declare that the research was conducted in the absence of any commercial or financial relationships that could be construed as a potential conflict of interest.

## Publisher’s note

All claims expressed in this article are solely those of the authors and do not necessarily represent those of their affiliated organizations, or those of the publisher, the editors and the reviewers. Any product that may be evaluated in this article, or claim that may be made by its manufacturer, is not guaranteed or endorsed by the publisher.
