# Supplementary material for: P2Y6 receptor-mediated signaling amplifies TLR-induced pro-inflammatory responses in microglia
Source: Front Immunol. 2022 Sep 20;13:967951. doi: 10.3389/fimmu.2022.967951 (PMC9531012; doi:10.3389/fimmu.2022.967951)
Supplement: Supplementary file 1 [file DataSheet_1.docx]

Supplementary Material

P2Y6 receptor-mediated signaling amplifies TLR-induced pro-inflammatory responses in microglia

Raissa Timmerman^1^, Ella A. Zuiderwijk-Sick^1^, Jeffrey J. Bajramovic^1, *^

^1^Alternatives Unit, Biomedical Primate Research Centre, Lange Kleiweg 161, 2288 GJ Rijswijk, The Netherlands

*** Correspondence:**Jeffrey J. Bajramovic
J.J.Bajramovic@uu.nl

**Fig. S1 Effects of MRS2578 on the viability of bone marrow-derived macrophages and microglia.** Bone marrow-derived macrophages (BMDM) and microglia were pre-incubated for 1 h with 0.2, 1, 5 or 25 µM MRS2578. Subsequently cells were stimulated with 10 µg/mL TLR4 agonist uLPS for 16 h. DMSO and unstimulated conditions were also included as a control. **A)** Images showing Hoechst+ and ethidium homodimer-1 (EthD-1)+ BMDM and microglia exposed to DMSO, 5 µM MRS2578 or 25 µM MRS2578 in the absence or presence of 16 h exposure to 10 µg/mL uLPS. Scale bars are 25 µm **B)** Cell viability was analyzed in 25 random fields of view. EthD-1+ nuclei were normalized to the number of Hoechst+ cells (n=1).

**Fig. S2 P2RY6 mRNA expression levels in microglia and BMDM.** P2RY6 mRNA expression levels in primary microglia and bone marrow-derived macrophages were normalized to housekeeping gene ACTB. Symbols represent different donors, n=5, paired t-test on log-transformed data.

**Fig. S3 Fold change cytokine gene expression after uLPS stimulation.** Microglia were stimulated with 10 µg/mL uLPS. After 16 h incubation the gene expression of IL-6, IL-8, IL-12p40, TNF-α and IL-1α was analyzed. The graph shows the fold change gene expression after uLPS stimulation normalized to its unstimulated control. Gene expression was normalized to ACTB. n=5, each symbol represents a donor.

**Fig. S4 P2RY6 silencing in primary microglia.** Primary microglia were transfected with either 72 nM non-targeting control siRNA (siNT) or 72 nM P2RY6-targeting siRNAs (siP2RY6). 40 h after transfection, P2RY6 mRNA levels were analyzed. P2RY6 expression levels were normalized to ACTB. Each symbol represents a donor. n=5, paired t-test on log-transformed data, * p < 0.05, **** p < 0.001.

**Fig. S5 Evaluation of apoptosis by use of TUNEL assay. A)** Images showing DAPI+ and TUNEL+ microglia exposed to DMSO or 5 µM MRS2578, in the absence or presence of 10 µg/mL uLPS. DNase I treatment was included in the TUNEL assay protocol as positive control (upper panels). White arrows indicate TUNEL+/DAPI+ microglia. Scale bars are 50 µm. **B)** Apoptosis was analyzed in 50 random fields of view from two technical replicates in total. TUNEL+ nuclei were normalized to the number of DAPI+ cells. Symbols represent different donors, n=3, one-way ANOVA.

| **IL-6** | **BMDM** | | **Microglia** | |
| --- | --- | --- | --- | --- |
|  | LogFC | SD | LogFC | SD |
| TLR1/2 | 0,09791423 | 1,14735583 | *-0,9316018* | *0,3978378* |
| TLR2/4 | -0,1022535 | 0,24505571 | -0,6856904 | 0,49332544 |
| TLR4 | -0,2839329 | 0,28119094 | *-0,9622986* | *0,353432* |
| TLR5 | -0,2367002 | 0,3085705 | -1,9566037 | 1,58541367 |
| TLR8 | *-0,2053414* | *0,0451123* | *-0,8028271* | *0,437632* |
|  |  |  |  |  |
| **IL-8** | **BMDM** | | **Microglia** | |
|  | LogFC | SD | LogFC | SD |
| TLR1/2 | 0,88063173 | 1,03529491 | *-0,4312042* | *0,1057365* |
| TLR2/4 | 0,74541259 | 0,29120097 | *-0,4653554* | *0,2019728* |
| TLR4 | *0,1979982* | *0,3466912* | -0,717875 | 0,76932858 |
| TLR5 | -0,0834408 | 0,19636463 | *-0,5240429* | *0,3065974* |
| TLR8 | -0,1039932 | 0,0475027 | *-0,6457286* | *0,2228286* |
|  |  |  |  |  |
| **IL-12p40** | **BMDM** | | **Microglia** | |
|  | LogFC | SD | LogFC | SD |
| TLR1/2 | -1,2139681 | 1,26949067 | *-2,5474273* | *1,6742343* |
| TLR2/4 | -0,1139073 | 0,29014978 | *-1,7964967* | *0,3912375* |
| TLR4 | 0,00396496 | 0,33236835 | *-1,5806403* | *0,4409508* |
| TLR5 | *-0,5793699* | *0,2852369* | *-1,5779896* | *0,6216272* |
| TLR8 | -0,4155901 | 0,39706591 | *-1,5807464* | *0,6228426* |
|  |  |  |  |  |
| **TNF-⍺** | **BMDM** | | **Microglia** | |
|  | LogFC | SD | LogFC | SD |
| TLR1/2 | -0,297644 | 1,32440258 | -0,7946214 | 1,35620328 |
| TLR2/4 | -0,2985793 | 0,57218531 | -1,0833412 | 2,08604117 |
| TLR4 | -0,652677 | 0,92778218 | -1,698678 | 2,51624274 |
| TLR5 | -0,2289576 | 1,19329679 | *-1,3441206* | *0,3835932* |
| TLR8 | 0,35582761 | 1,85515577 | *-1,5533014* | *0,7557958* |

**Table S1.** Mean log2 fold change (LogFC) cytokine production values of bone marrow-derived macrophages (BMDM) and microglia exposed several TLR agonists, in the presence versus absence of P2RY6 antagonist MRS2578 (raw data are presented in **Fig 1A** and **Fig 1B**). Positive values refer to an upregulation of the cytokine production in combination with P2RY6 antagonist, whereas negative values refer to a downregulation of the cytokine production in combination with P2RY6 antagonist. Values in italic represent values that are statistically significant in the presence of P2RY6 antagonist.

|  |  |  |  |  |  |  |
| --- | --- | --- | --- | --- | --- | --- |
| **IL-6/ACTB** | **R00043** | **R03098** | **R02046** | **R02008** | **R13169** | **R18015** |
| (-) | 0,00003546 | 0,00809091 | 0,000119649 | 0,000259471 | 0,000025070 | 0,000064453 |
| PAM3CSK4 | 0,00505291 | 0,09402169 | 0,001432174 | 0,010555881 | 0,015537682 | 0,017049217 |
| MRS2578 + PAM3CSK4 | 0,00354260 | 0,03986960 | 0,001986299 | 0,013266050 | 0,009149290 | 0,014251765 |
| LPS | nd | nd | 0,002010874 | 0,007635992 | 0,039023494 | 0,006479842 |
| MRS2578 + LPS | nd | nd | 0,000586456 | 0,002503999 | 0,008138432 | 0,006214852 |
| uLPS | 0,00215710 | nd | 0,000387123 | 0,002385828 | 0,005918083 | 0,007044204 |
| MRS2578 + uLPS | 0,00151713 | nd | 0,000349639 | 0,001561034 | 0,004486201 | 0,004105388 |
| Flagellin | nd | nd | 0,000336297 | 0,001106794 | 0,004612060 | 0,005982744 |
| MRS2578 + Flagellin | nd | nd | 0,000236460 | 0,001632871 | 0,003964269 | 0,004774585 |
| CL075 | 0,01313472 | nd | 0,005542869 | 0,013215434 | 0,059942860 | 0,041287295 |
| MRS2578 + CL075 | 0,00910964 | nd | 0,004138057 | 0,011380382 | 0,060751683 | 0,046098542 |
| **IL-8/ACTB** | **R00043** | **R03098** | **R02046** | **R02008** | **R13169** | **R18015** |
| (-) | 0,00676343 | 0,46428291 | 0,042480264 | 0,026033796 | 0,006932328 | 0,017944201 |
| PAM3CSK4 | 0,14406385 | 3,07991283 | 0,320423221 | 0,098081411 | 0,258565436 | 0,215436542 |
| MRS2578 + PAM3CSK4 | 0,18724558 | 1,52682675 | 0,354203953 | 0,355547152 | 0,243773833 | 0,141261101 |
| LPS | nd | nd | 0,425017396 | 0,304688363 | 4,039544043 | 0,024601169 |
| MRS2578 + LPS | nd | nd | 0,136703308 | 0,401130869 | 0,995533895 | 0,027736159 |
| uLPS | 0,11494742 | nd | 0,119011188 | 0,238626146 | 0,216448907 | 0,095866927 |
| MRS2578 + uLPS | 0,11206407 | nd | 0,120308380 | 0,260026965 | 0,177714434 | 0,092739914 |
| Flagellin | nd | nd | 0,147698854 | 0,235887710 | 0,546807580 | 0,036537710 |
| MRS2578 + Flagellin | nd | nd | 0,149964051 | 0,132458361 | 0,345863353 | 0,037237714 |
| CL075 | 0,32821085 | nd | 0,382848136 | 0,374025668 | 3,569052413 | 0,156891751 |
| MRS2578 + CL075 | 0,27242166 | nd | 0,376768576 | 0,657559471 | 3,339620871 | 0,252047561 |
| **IL-12p40/ACTB** | **R00043** | **R03098** | **R02046** | **R02008** | **R13169** | **R18015** |
| (-) | 0,00057756 | 0,00017687 | 0,000749509 | 0,000061276 | 0,000086144 | 0,000322478 |
| PAM3CSK4 | 0,01002188 | 0,00268937 | 0,021635986 | 0,005355699 | 0,014873942 | 0,051242818 |
| MRS2578 + PAM3CSK4 | 0,00446097 | 0,00153295 | 0,040808361 | 0,003473477 | 0,004924395 | 0,016804548 |
| LPS | nd | nd | 0,057416466 | 0,026742094 | 0,305821295 | 0,048845037 |
| MRS2578 + LPS | nd | nd | 0,006497211 | 0,007109192 | 0,023801902 | 0,028772254 |
| uLPS | 0,01257565 | nd | 0,017749187 | 0,008199787 | 0,054242274 | 0,050325566 |
| MRS2578 + uLPS | 0,00829204 | nd | 0,005951416 | 0,003096993 | 0,011150957 | 0,018128453 |
| Flagellin | nd | nd | 0,010431962 | 0,004940737 | 0,019247820 | 0,038043230 |
| MRS2578 + Flagellin | nd | nd | 0,008821423 | 0,002661590 | 0,007176620 | 0,013740936 |
| CL075 | 0,21011839 | nd | 0,108311782 | 0,077890833 | 0,224764040 | 0,350902075 |
| MRS2578 + CL075 | 0,06308783 | nd | 0,075680581 | 0,029184517 | 0,135129357 | 0,233385127 |
| **TNFA/ACTB** | **R00043** | **R03098** | **R02046** | **R02008** | **R13169** | **R18015** |
| (-) | 0,00001392 | 0,00083630 | 0,000037959 | 0,000010851 | 0,000063357 | 0,000053520 |
| PAM3CSK4 | 0,00051133 | 0,00276131 | 0,000187339 | 0,000320112 | 0,000561454 | 0,000323389 |
| MRS2578 + PAM3CSK4 | 0,00037943 | 0,00061963 | 0,000203916 | 0,000364483 | 0,000506409 | 0,000231879 |
| LPS | nd | nd | 0,000318122 | 0,000106244 | 0,015634313 | 0,000300679 |
| MRS2578 + LPS | nd | nd | 0,000096600 | 0,000139676 | 0,000749734 | 0,000240245 |
| uLPS | 0,00020675 | nd | 0,000169584 | 0,000074032 | 0,000293562 | 0,000306923 |
| MRS2578 + uLPS | 0,00022255 | nd | 0,000125898 | 0,000091913 | 0,000248107 | 0,000204246 |
| Flagellin | nd | nd | 0,000073184 | 0,000058024 | 0,000238400 | 0,000168100 |
| MRS2578 + Flagellin | nd | nd | 0,000149208 | 0,000040544 | 0,000273863 | 0,000223089 |
| CL075 | 0,08710478 | nd | 0,000573749 | 0,000480253 | 0,002418964 | 0,001254224 |
| MRS2578 + CL075 | 0,00047618 | nd | 0,000343933 | 0,000251236 | 0,001218810 | 0,000866307 |
| **IL-1α/ACTB** | **R00043** | **R03098** | **R02046** | **R02008** | **R13169** | **R18015** |
| (-) | 0,00009837 | 0,04198078 | 0,000196243 | 0,000062831 | 0,000020978 | 0,000055438 |
| PAM3CSK4 | 0,02569045 | 0,325775226 | 0,046508566 | 0,024183643 | 0,016242005 | 0,032641548 |
| MRS2578 + PAM3CSK4 | 0,01698758 | 0,071541166 | 0,033286588 | 0,013321629 | 0,011561838 | 0,008389118 |
| LPS | nd | nd | 0,064572037 | 0,037018368 | 0,090078563 | 0,017819370 |
| MRS2578 + LPS | nd | nd | 0,013610010 | 0,013124692 | 0,025125642 | 0,006772212 |
| uLPS | 0,02553467 | nd | 0,021496998 | 0,017350903 | 0,014089178 | 0,012322661 |
| MRS2578 + uLPS | 0,01332532 | nd | 0,011156551 | 0,004987339 | 0,005885766 | 0,004338258 |
| Flagellin | nd | nd | 0,022376019 | 0,011896503 | 0,017255870 | 0,035371350 |
| MRS2578 + Flagellin | nd | nd | 0,011882614 | 0,004418004 | 0,004991544 | 0,016246457 |
| CL075 | 0,04094127 | nd | 0,050298625 | 0,043691685 | 0,113355118 | 0,077666500 |
| MRS2578 + CL075 | 0,02697493 | nd | 0,034084199 | 0,031311564 | 0,055822039 | 0,065235912 |

**Table S2.** Cytokine mRNA expression levels normalized to ACTB. Microglia were exposed to different TLR ligands for 16 h, with or without 1 h pre-incubation of the P2RY6 antagonist MRS2578 (5 µM). TLR ligands used are 1 µg/mL Pam_3_CSK_4_ (TLR1/2), 10 ng/mL LPS (TLR2/4), 10 ng/mL uLPS (TLR4), 100 ng/mL Flagellin (TLR5) and 1 µg/mL CL075 (TLR8).

|  | **R07110** | | **R14143** | | **R12016** | | **R02046** | | **R02008** | |
| --- | --- | --- | --- | --- | --- | --- | --- | --- | --- | --- |
| **IL-6** | pg/mL | SD | pg/mL | SD | pg/mL | SD | pg/mL | SD | pg/mL | SD |
| (-) | 452 | - | 513 | 11 | bd | bd | bd | bd | 84 | 118 |
| 5 µM MRS2578 | 489 | 109 | 157 | - | bd | bd | bd | bd | bd | bd |
| 10 ug/mL uLPS | 3520 | 523 | 2055 | 541 | 10596 | 869 | 2216 | 587 | 1481 | 787 |
| 5 µM MRS2578 + uLPS | 1756 | 387 | 1587 | 541 | 2185 | 663 | 910 | 168 | 144 | 78 |
| 1 µM MRS2578 + uLPS | 2598 | 142 | 1868 | 251 | 8376 | 147 | 1914 | 712 | 1888 | 338 |
| 0.2 µM MRS2578 + uLPS | 3127 | 63 | 2219 | 90 | 8642 | 213 | 2249 | 1217 | 1075 | 622 |
| **IL-8** |  |  |  |  |  |  |  |  |  |  |
| (-) | 19461 | - | 50277 | 18472 | 3151 | 602 | bd | bd | 98747 | 28861 |
| 5 µM MRS2578 | 19760 | 27945 | 114005 | 52059 | 14790 | 2208 | bd | bd | 121840 | 28101 |
| 10 ug/mL uLPS | 186077 | 45736 | 256898 | 96282 | 237774 | 12445 | 143980 | 8400 | 283492 | 107849 |
| 5 µM MRS2578 + uLPS | 144730 | 10420 | 289158 | 108317 | 73410 | 29306 | 102730 | 49935 | 184675 | 30380 |
| 1 µM MRS2578 + uLPS | 188124 | 2894 | 330719 | 57937 | 220600 | 12645 | 230110 | 63003 | 449440 | 6835 |
| 0.2 µM MRS2578 + uLPS | 201428 | 3763 | 305387 | 51779 | 225851 | 21678 | 250240 | 18667 | 352771 | 111647 |
| **IL-12p40** |  |  |  |  |  |  |  |  |  |  |
| (-) | bd | bd | bd | bd | bd | bd | 86 | 31 | 52 | 7 |
| 5 µM MRS2578 | bd | bd | bd | bd | bd | bd | 74 | 3 | bd | bd |
| 10 ug/mL uLPS | 680 | 83 | 2396 | 584 | 10153 | 997 | 2471 | 224 | 304 | 35 |
| 5 µM MRS2578 + uLPS | 302 | 261 | 1796 | 1165 | 1453 | 44 | 1337 | 332 | 78 | 14 |
| 1 µM MRS2578 + uLPS | 456 | 7 | 2254 | 350 | 9125 | 1244 | 2578 | 267 | 507 | 95 |
| 0.2 µM MRS2578 + uLPS | 961 | 64 | 1670 | 960 | 8194 | 970 | 1407 | 17 | 467 | 117 |
| **TNF-α** |  |  |  |  |  |  |  |  |  |  |
| (-) | bd | bd | bd | bd | 113 | 160 | bd | bd | bd | bd |
| 5 µM MRS2578 | bd | bd | bd | bd | bd | bd | bd | bd | bd | bd |
| 10 ug/mL uLPS | 419 | 136 | 1313 | 416 | 7928 | 1406 | 165 | 161 | 494 | 418 |
| 5 µM MRS2578 + uLPS | 408 | 193 | 1262 | 927 | 791 | 76 | 440 | 57 | 65 | 0 |
| 1 µM MRS2578 + uLPS | 281 | 33 | 1573 | 189 | 4292 | 1207 | 214 | 294 | 504 | 106 |
| 0.2 µM MRS2578 + uLPS | 560 | 59 | 693 | 508 | 4326 | 95 | 318 | 36 | 684 | 159 |

**Table S3.** IL-6, IL-8, IL-12p40 and TNF-α levels in picograms per milliliter per donor. The detection limits for IL-6, IL-12p40 and TNF-α were 8 pg/mL, the detection limit for IL-8 was 31 pg/mL. bd = below detection, nd = no data, - = no SD due to 1 measurement.

| **P2RY6/ACTB** | **R01085** | **R06050** | **R14143** | **R15150** | **R06054** |
| --- | --- | --- | --- | --- | --- |
| (-) | 0,002074117 | 0,003942705 | 0,001432201 | 0,001249353 | 0,005158663 |
| 72 nM siNT | 0,001648237 | 0,002442381 | 0,001300708 | 0,000634292 | 0,003631725 |
| 72 nM siP2RY6 | 0,000214067 | 0,000228962 | 0,000078663 | 0,000073152 | 0,000570325 |
| **IL-6/ACTB** | **R01085** | **R06050** | **R14143** | **R15150** | **R06054** |
| (-) | 0,000025281 | 0,000065677 | 0,000012724 | 0,000039156 | 0,000143823 |
| (-) + uLPS | 0,017661622 | 0,009952204 | 0,085580582 | 0,029290366 | 0,016208134 |
| 72 nM siNT + uLPS | 0,011331460 | 0,014389294 | 0,550316899 | 0,039067413 | 0,013647038 |
| 72 nM siP2RY6 + uLPS | 0,007886920 | 0,002817470 | 0,028615537 | 0,016457138 | 0,009726473 |
| **IL-12p40/ACTB** | **R01085** | **R06050** | **R14143** | **R15150** | **R06054** |
| (-) | 0,001737157 | 0,000728342 | 0,000085712 | 0,000408871 | 0,000181550 |
| (-) + uLPS | 0,026604241 | 0,028845215 | 0,057045250 | 0,058016014 | 0,037350047 |
| 72 nM siNT + uLPS | 0,015142563 | 0,019957635 | 0,110589963 | 0,063076055 | 0,035672200 |
| 72 nM siP2RY6 + uLPS | 0,010763012 | 0,008455282 | 0,043292286 | 0,039226616 | 0,021020553 |
| **IL-1α/ACTB** | **R01085** | **R06050** | **R14143** | **R15150** | **R06054** |
| (-) | 0,000043445 | 0,000049981 | 0,000011743 | 0,000070075 | 0,000010360 |
| (-) + uLPS | 0,035587229 | 0,121438298 | 0,107656972 | 0,096557961 | 0,075797481 |
| 72 nM siNT + uLPS | 0,037694923 | 0,089814780 | 0,131282181 | 0,046911438 | 0,029931014 |
| 72 nM siP2RY6 + uLPS | 0,033539940 | 0,045640298 | 0,038851538 | 0,036757301 | 0,028843083 |

**Table S4.** P2RY6, IL-6, IL-12p40 and IL-1α mRNA expression levels normalized to ACTB. Microglia were untransfected (-), transfected with 72 nM non-targeting control siRNA (siNT) or transfected with 72 nM P2RY6-targeting siRNAs (siP2RY6). 24 h after transfection, microglia were stimulated with 10 µg/mL uLPS and mRNA expression levels were analyzed after 16 h.

|  | **siP2RY6 transfected microglia vs.**  **Untransfected microglia** | | **siP2RY6 transfected microglia vs.**  **siNT transfected microglia** | |
| --- | --- | --- | --- | --- |
|  | LogFC | SD | LogFC | SD |
| **IL-6** | *-1,226525167* | *0,468516752* | -1,775313365 | 1,584055946 |
| **IL-12p40** | *-0,973581375* | *0,562253274* | *-0,906568382* | *0,371105356* |
| **IL-1α** | *-1,151001989* | *0,596477388* | -0,661417347 | 0,708394675 |

**Table S5.** Mean log2 fold change (LogFC) cytokine mRNA expression values of microglia transfected with P2RY6-targeting siRNAs (siP2RY6) versus untransfected microglia, and versus microglia transfected with non-targeting control siRNAs (siNT) (raw data are presented in **Fig 2C**). The negative values refer to a downregulation of the cytokine mRNA expression levels in microglia transfected with P2RY6-targeting siRNAs. Values in italic represent values that are statistically significant.

**Table S6.** Excel file with the Ensemble IDs, log fold changes (LogFC), false discovery rates (FDR), gene names, counts per million (CPMs) values and log CPM values of the 302 differentially expressed genes (FDR < 0.05) between 10 µg/mL uLPS and 10 µg/mL uLPS + 5 µM P2RY6 antagonist MRS2578 stimulated microglia.

**Table S7.** Excel file with the differentially expressed genes (DEG) associated with the biological processes displayed in Fig. 3C

**Table S8.** Excel file with the differentially expressed genes (DEG) associated with the transcription factor targets displayed in Fig. 3E

**Table S9.** Excel file with the differentially expressed genes (DEG) associated with the pathways displayed in Fig. 3F
